# Global meridional eddy heat transport inferred from Argo and altimetry observations

**DOI:** 10.1038/s41598-018-38069-2

**Published:** 2019-02-04

**Authors:** Bowen Sun, Chuanyu Liu, Fan Wang

**Affiliations:** 1 CAS Key Laboratory of Ocean Circulation and Waves, Institute of Oceanology, Chinese Academy of Sciences (IOCAS), Qingdao, China; 2 University of Chinese Academy of Sciences, Beijing, China; 3 Marine Dynamic Process and Climate Function Laboratory, Pilot National Laboratory for Marine Science and Technology (Qingdao) (QNLM), Qingdao, China; 4 Center for Ocean Mega-Science, Chinese Academy of Sciences, Qingdao, China

## Abstract

Proportion and pathways of the eddy-induced heat transport are critical in maintaining world’s ocean and climate states. However, an observation-based three-dimensional picture of how oceanic eddies contribute to the global heat transport is yet not quantitatively specified, particularly due to insufficiency of data. Here, using refined methods, we have achieved this goal by analyzing millions of high-quality Argo hydrographic profiles and high-resolution satellite altimetric data. We first presented the spatial differences of individual eddies by reconstructing 254 representative eddies all over the ocean, and then calculated heat fluxes associated with eddies in 5° × 5° boxes. It is revealed that all parameters of eddies vary significantly with both latitudes and longitudes, which is crucial in yielding spatially varying heat fluxes and transports. The eddies not only transport heat towards high latitudes (down-gradient), but also towards low latitudes (up-gradient), particularly at subsurface layers of mid-latitude northern Pacific Ocean and low-latitude Atlantic Ocean. The eddy heat transport is mainly confined in the upper 1000 m of the western part and mid-latitudes of the world’s ocean basins, coinciding with maximum meridional temperature gradients. It peaks at 0.8 PW and 0.3 PW (1 PW = 10^15^ W) at 45°S and 35°N, respectively, stronger than previous estimates based on model results, and accounts for about one half and one third of the estimated total oceanic heat transport at the same latitudes, respectively. In any location except for the areas associated with the Antarctic Circumpolar Current, the eddy stirring component is distinctly (1–10 times) larger than the eddy trapping component.

## Introduction

Through transporting large amounts of heat from tropics to high-latitudes and hence impacting ocean’s heat storage, sea-level rise and air-sea fluxes, the oceans have the ability to redistribute the Earth’s surface temperature, and act as an essential regulator of climate. Model simulations have suggested that, without poleward oceanic heat transport, the polar regions would cool significantly following with equatorward spreading of polar caps and eventual freezing allover of the Earth^1^. The oceans transport heat mainly through two mechanisms: heat advection by large scale circulation and heat fluxes due to mesoscale eddies. The momentous contribution of oceanic eddies has been widely acknowledged particularly from regional studies; however, a global picture of their effect could only be derived yet from outputs of eddy resolving ocean general circulation models^2–4^. Those results showed that in a number of locations, such as the tropics, the Southern Ocean and the Kuroshio Current, the eddy heat transport accounts for a considerable portion of the total time-mean heat transport^2,3^; in particular, the eddies can transport heat in a manner that either partially compensates or offsets for the heat transport from the large scale circulations^3^. However, two weaknesses might be faced with the model results-based estimation. One is that the estimates always are based on covariance between velocity and temperature anomalies in an Eulerian framework, where the anomalies may include signals of other than eddies; the other is that only a few of the high-resolution numerical models has observational constraints^3^ and many of them suffer from biases in modeling instantaneous eddies. Therefore, the model based eddy heat fluxes, particularly in magnitude, need to be justified by observations in order to evaluate eddy’s role in ocean heat transport.

Estimates of eddy heat transport based on reconstructed 3D eddies were carried out recently. For example, refs^5–7^ made estimates by tracking number of Argo floats, ref.^8^ used high-resolution transaction observations of sea water, and ref.^9^ used Argo profiles in specific regions of the world’s oceans. Results showed that, in the southern Atlantic (25°−35°S)^7^, the estimated mean meridional heat flux along a single eddy was about 0.027 PW for the mean eddy lifetime and 0.062 PW annually. In the northern Pacific (equatorward of 45°N)^5,8^, the zonally-integrated poleward heat transport associated with the Kuroshio Extension and the subtropical countercurrent (STCC) is at a level of 0.1 PW, half of that in the simulations^3^. It becomes clear that such estimates were conducted only with limited synoptic hydrographic data and/or in limited regions. Such estimates on the one hand demonstrate the considerable portion the eddies provide to the total oceanic heat transport (at *O*(1) PW)^10^, and the noticeable deviations from numerical models as well, but also imply potentially importance of eddy heat transport in many other regions of the world’s oceans, which, however, remain poorly identified and require to be figured out. Eddy heat transport was also estimated based on sea surface altimetry/temperature data^11,12^, but was confined to the mixed layer and specific regions. Exceptions include refs^13,14^, who estimated global eddy heat transport. However, ref.^13^ conducted the estimation in an Eulerian framework without considering heat flux contributed by individual eddies’ movement. ref.^14^ had considered movements of the individual eddies; however, they estimated only the trapping component of the eddy heat transport, without considering the stirring component.

Reference^14^ signifies one of the two mechanisms that eddy induces heat fluxes and heat transport. In addition to the commonly recognized mechanism that eddies induce heat fluxes by stirring local background isotherms via rotating, ref.^14^ illustrated that eddies can also induce heat fluxes by trapping and transporting water with certain temperature in their cores^14,15^ due to nonlinearity^16,17^. However, questions are raised from recognition of the trapping component. Firstly, the proportion of the two components are not clearly determined yet, and under debate with respect to their importance: ref.^12^ argued that the stirring component matches the total eddy fluxes well as indicated by simulations in the Southern Ocean; ref.^14^, by contrary, argued that it is the trapping component that mainly constitutes the eddy heat transport. Secondly, the trapping component was not represented in the coarse resolution ocean/climate models yet. The stirring component is usually believed to result in down-gradient heat fluxes and has long been represented in Fickian scheme and always treated with a constant mixing coefficient^18,19^ (The down-gradient hypothesis and constant coefficient treatment were challenged by some studies^20^, and they were not justified in direct eddy fluxes estimations). By contrary, to our knowledge, the trapping process has even not been parameterized yet; consequently, it remains unclear if missing of it may lead to great model drifts.

Therefore, a global-scale distribution of eddy heat fluxes/transports, of both stirring and trapping components, is important not only for understanding the dynamics of oceanic eddies, but also for the purpose of identifying the high resolution models’ performance, and amending/proposing parameterizations for eddies’ effect in the coarse resolution models. The present study is thus to map the 3D global-scale distributions. To do this, the spatial variability of eddies are identified first.

## Spatial Variations of Composite Eddies

In order to highlight the associativity of the trapping and stirring components of eddy heat fluxes with variety of eddy structures, we first reconstructed full-structure eddies with associated temperature anomalies, rotational velocities and their average movement speeds following pioneer methods^5,14,15^. This is conducted by compositing sufficient Argo hydrographic profiles that are concurrently captured by sea surface eddies in selected regions (Fig. S1). The regions are selected such that on the one hand each of them is large enough to encompass sufficient Argo profiles for eddy composition, on the other hand they are widely distributed on the world’s oceans to represent spatial variation of the eddies. Accordingly, we divided the global oceans into 58 consecutive 5° wide latitudinal bands, each of which extends from the western to the eastern boundaries of corresponding ocean basins. Then, we further divided each latitudinal band into 1~5 size-varying sub-regions subject to amount of eddy-captured Argo profiles. The minimum limit of profiles for a single sub-region is set to 3000. This is sufficient for eddy composition. Only in limited areas of the Southern Ocean, the sub-region is slightly meridionally extended and overlapped to fulfill the above condition. Over all, the ocean basins were divided into 127 sub-regions (Fig. S1). Each sub-region produces both a composite warm (anti-cyclonic) eddy and a composite cold (cyclonic) eddy (see Method). In this way, the three-dimensional average eddy structures are reconstructed, which is different from horizontal or vertical distribution of eddy’s temperature, salinity and other properties obtained by limited observational data^21–23^ or reanalysis data^24^.

**Figure 1:**
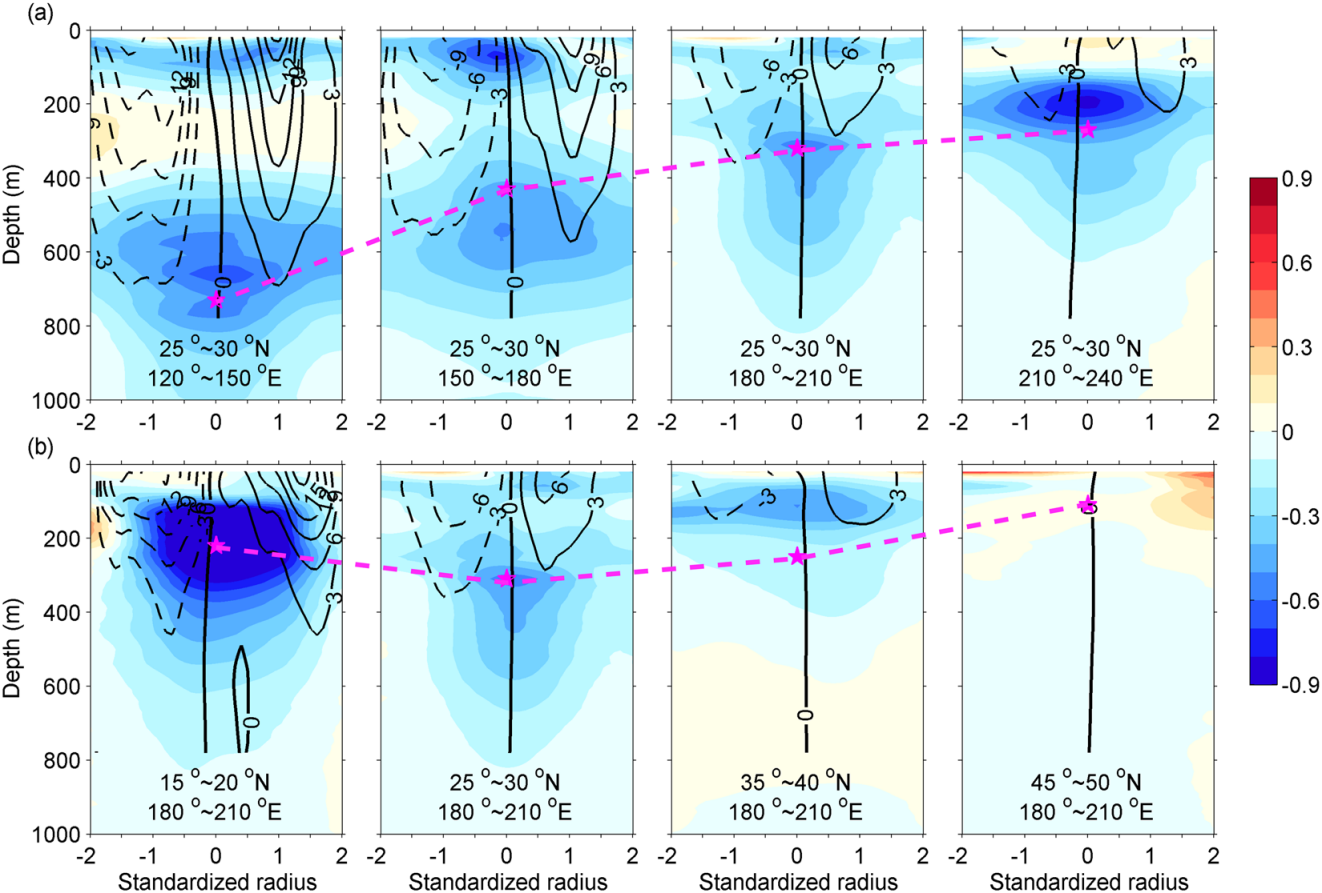
Spatial variation of cold-core (cyclonic) eddies in the northern Pacific. (**a**), Cold-core eddies composited in sub-regions along a meridional band: 25°~30°N; (**b**), the same as (**a**), but along a zonal band: 180°~210°E. Boarders of each sub-region are denoted by insets. Color shading and contours denote anomalous temperature (unit: °C) and meridional geostrophic rotation velocity (unit: cm/s), respectively. Shown are along the west-east section across eddy center. Dashed magenta line connects the eddy trapping depth of each sub-region denoted by Asterisk.

The properties of the composite eddies, including their size, intensity, structure and movement speed, show both latitude and longitude dependency. For illustration, Fig. 1 presents sections of eight composite eddies that are obtained in the northern Pacific (and for reference, Fig. S1a presents the whole-structures for one of them). The eddies are intensified towards west of the ocean basins: with eddy size (radius) growing from 95 km in the eastern interior (210°−240°E) to 108 km in the western regions (120°−150°E), eddy intensity (maximum rotation velocity) increasing from ~5 cm/s to ~20 cm/s, and eddy trapping depth (see Method) deepening from ~270 m to ~720 m (Fig. 1a). This westward intensification feature is supported by the conclusion of ref.^25^ in the similar region, and stands for the same trend in other latitudes and other ocean basins, except for the tropics and the Southern Ocean, which is not bounded by meridional boundaries (Figs S3 and S4c). In the interior of the northern Pacific Ocean, the eddies are most amplified in the subtropical countercurrent (STCC) band (15°−20°N; Fig. 1b).

From the view point of anomalous temperatures, the eddies also vary significantly with both latitudes and longitudes. In general, in an ocean state with poleward temperature gradient, a cold eddy can be separated into two components: a nearly symmetric cold component (monopole) located at the eddy center due to divergence, and a nearly anti-symmetric component (dipole) on the eastern and western sides due to horizontal advection of the sea surface temperature (SST) field^9,11,12^. The two components can be loosely inferred from Fig. 1. It shows that the dipole, if it exists, seems to be discernable only in the upper 200 m layers^5,9^. The monopole, on the other hand, is significant, and may be intensified at different layers: surface layers (e.g. in the middle Pacific), shallow subsurface layers (e. g, in the northeastern Pacific) and deep subsurface layers (e. g., in the western Pacific). Moreover, sandwich core structure (cold-warmer-cold cores) is found in this region and in some other regions as well. For example, the eddy of the subtropical western Pacific (e. g., the first panel of Fig. 1a) has one cold core at surface layers (0–200 m), another cold core in the main thermocline (500–800 m) and a warm layer in-between. From the lower cold core downward, the temperature anomaly has little vertical phase tilt but decreases dramatically to a negligible value above ~1000 m. Note that neither the temperature anomalies nor the rotation velocity are strictly symmetric about the eddy core; the isolines of the two properties do not align with each other, either. The asymmetry is crucial for the eddy to induce net heat fluxes^8^.

The meridional deflection along with the westward movement of eddies, i.e., the meridional movement of the eddies, are responsible for the trapping component of the meridional heat fluxes. The meridional movement speeds show pretty similar patterns for both anti-cyclonic and cyclonic eddies. They are larger in tropical, sub-polar and western boundary regions (>2 cm/s), though they are one order smaller than the rotational velocity (Figs S3 and 4; also in refs^13,17^). The meridional directions are generally equatorward in the subtropics and poleward in the tropics. Exceptions include the Pacific cold tongue region and the tropical Indian Ocean, where they are southward. In addition, in the Southern Ocean, the anti-cyclonic eddies tend to move poleward while the cyclonic eddies tend to move equatorward.

Given the prominent spatial variation of eddy parameters, it’s naturally inferred that eddy heat fluxes in the world’s oceans will show spatial variations.

## Vertical and Meridional Variations of Meridional Eddy Heat Fluxes

**Figure 2:**
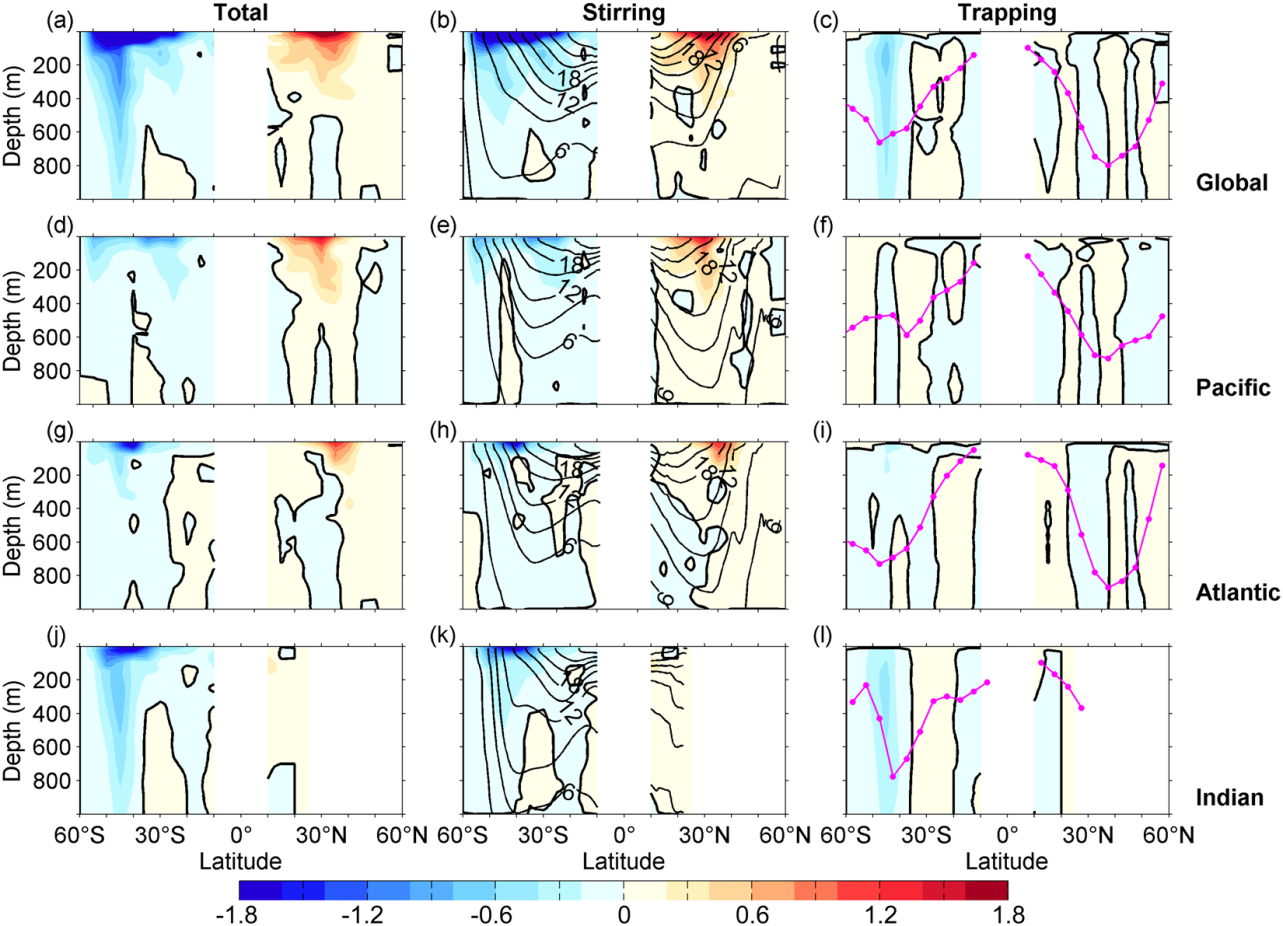
Latitude-depth distribution of zonally-integrated meridional eddy heat flux. Columns are for total (left; total = stirring + trapping), stirring (middle), and trapping (right) component of eddy heat fluxes (color; unit: 10^12^ W/m), respectively, while rows are for the Global, the Pacific, the Atlantic and the Indian Oceans, respectively. Local occurrence frequency and intensity of eddies are taken into account (see Method). Contours in the middle column are for climatological isotherms (CI: 3 °C); magenta line is for the zonal mean of the eddy trapping depth.

With the parameters of each composite eddy, the resultant trapping and stirring components of eddy meridional heat fluxes are readily quantifiable (see Method and Fig. S2b). They show two pronounced characteristics. Firstly, composite eddy usually has much larger stirring flux than trapping flux in most layers, primarily due to one order larger meridional rotate velocity (than the meridional movement speed; Figs S3c and S4a,b). Secondly, stirring flux is surface intensified and usually confined above 1000 m (while the trapping component is confined above the shallower trapping depth, see Method). An important feature is that the directions of fluxes may alternate between northward and southward, which implies that the eddy heat fluxes are not only down-gradient of the isotherms (poleward), but also up-gradient (equatorward) at specific depths. This feature is found in previous study^4^ and is typical in the northern hemisphere, as shown in Fig. 2.

The 254 composite eddies enable us to produce a global map of eddy meridional heat fluxes (at 5° × 5° horizontal resolution, see Method). Figure 2 presents the dependency of them on both depth and latitude. It demonstrates again that, in the northern hemisphere, in the subtropics to tropics of the southern hemisphere, and in the entire Pacific Ocean, the total eddy heat fluxes are dominated by the stirring component; the trapping component is comparable to the stirring component only in the Antarctic Circumpolar Current (ACC) region. The maximum stirring flux is ~2 × 10^12^ W/m which takes place at the surfaces. The maximum trapping flux, however, is much smaller. It is only ~0.6 × 10^12^ W/m, and takes place at 40°S of the Indian Ocean with southward direction. The large difference in magnitude between the two components can be interpreted as below: anti-cyclonic and cyclonic eddies trap water with opposite temperature anomalies (Fig. S3a,b) but move at similar speeds and in similar directions (including in the meridional direction) in the majority of basins; consequently, they offset each other in trapping and transporting heat. In the ACC region, by contrary, the cyclonic and anti-cyclonic eddies seem to move oppositely in the meridional direction in many areas (Fig. S4) where the associated heat transports add up. Whereas, the stirring heat transport induced by anti-cyclonic and cyclonic eddies almost has the same spatial patterns and signs, which eventually enhances each other.

The stirring component displays significant vertical variations (Fig. 2, middle column). In a wide range of the northern Atlantic Ocean (10°−30°N), the heat flux, though weak, are equatorward in the subsurface layers. While model simulations do have found southward heat fluxes^2^ in the southern flank of the Gulf Stream, those in a vaster areas as revealed here need for explanation but exceed the scope of this study. In the southern hemisphere, a general poleward pattern is found in all the ocean basins; however, equatorward fluxes also show up in low-latitudes of the Indian and Atlantic oceans, and in the Pacific section of ACC.

It has been revealed in regional scale estimate, that eddies are confined above the thermocline^4^. The global scale of this feature is indicated in Fig. 2: the bottom of the eddies (inferred as the trapping depth; right column of Fig. 2) peaks at around 40° in both hemispheres (at ~900 m in the northern hemisphere and ~800 m in the southern hemisphere) and gradually shoals up both poleward (until 600 m in the southern hemisphere and 200 m in the northern hemisphere) and equatorward. The trapping depths are well close to the bottoms of the main thermocline (middle column of Fig. 2).

By contrary, the trapping fluxes show prominent meridional variations (Fig. 2, right column): equatorward and poleward heat fluxes alternate with latitudes. This pattern represents the latitudinal dependence of the meridional movement speed of the eddies (Fig. S4a,b). Note that, equatorward heat fluxes again are up-gradient of the temperature distribution, which needs to be represented in low resolution models.

## Horizontal Pathways of Meridional Eddy Heat Transport

**Figure 3:**
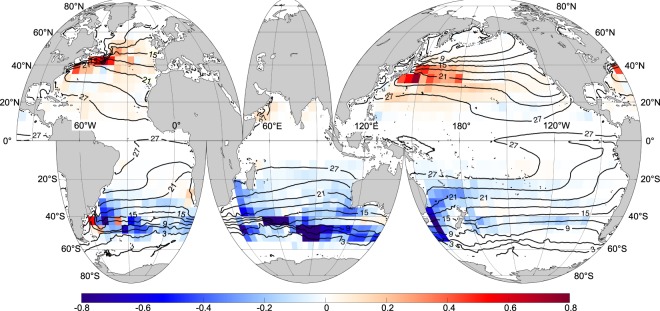
Horizontal distribution of depth-integrated meridional eddy heat transport. The color shows total (stirring + trapping) meridional eddy heat transport integrated zonally in 5° × 5° boxes (unit: 10^13^ W). Positive and negative values are for northward and southward transport, respectively. Black lines are for climatological isotherms (CI: 3 °C) derived from AMSR-E SST data.

The depth-integrated heat transports at each 5° × 5° box are presented (see Method) for the world’s oceans in Fig. 3. This figure shows that the meridional eddy heat transports are confined to certain latitudes, rather than consecutive from the equators to the poles. They are also confined in certain regions. Generally, the heat transport is high in the mid-latitudes of western part of the ocean basins, where the local maximum reaches nearly 0.1 PW at ~40° in both hemispheres. Taking the northern Pacific Ocean as an example, the transports occur between 20° and 40°N, but the largest transport only occurs between 30° and 40°N, and limited to west of the dateline. The locations of maximum heat transport are generally zonally aligned and are referred as heat convergent zones. The convergent zones include the western boundary currents and their extensions, the STCC of the Pacific and Indian Oceans, and the ACC as well. The heat convergence zones are also generally consistent with large SST gradients; poleward of the convergent zones the oceans are heated by eddies while equatorward of them the oceans are cooled, signifying eddies’ effect on ocean temperature’s maintenance, redistribution and exchange across isotherms.

## Total Meridional Eddy Heat Transport

**Figure 4:**
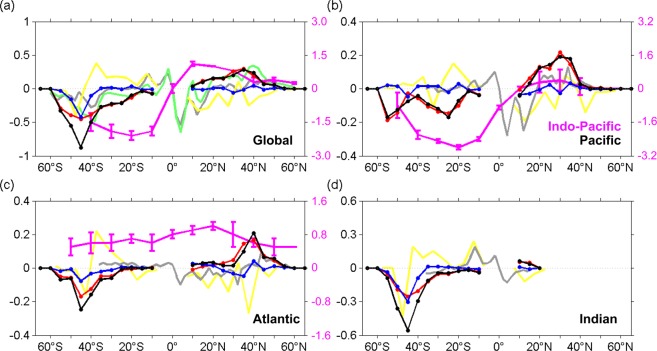
Latitude dependence of total meridional eddy heat transport. Zonally- and depth-integrated total (black lines), stirring (red lines) and trapping (blue lines) meridional eddy heat transport (unit: PW = 10^15^ W). (**a**) Global Ocean, (**b**) Pacific Ocean, (**c**) Atlantic Ocean and (**d**) Indian Ocean. The green line in (**a**) denotes the estimated heat transport from Griffies *et al*.’s model study (ref.^4^), and the gray lines are for that in Volkov *et al*.’s model study (ref.^3^); the yellow lines denote the trapping heat transport estimated by Dong *et al*. (ref.^14^) based on Argo data; the magenta lines are for total oceanic northward heat transport in Macdonald and Wunsch’s (ref.^10^). Note the different scale of this data; note that the magenta line in (**b**) is for Indo-Pacific Ocean.

Integrating both of the stirring and trapping components of the eddy heat transport at each 5° × 5° box zonally and vertically, the meridional distribution of the total meridional eddy heat transports were obtained (Fig. 4). The global eddy heat transport (black line) peaks at mid-latitudes: ~0.3 PW at ~35°N, and ~0.8 PW at ~45°S (Fig. 4a), and becomes nearly zero at tropics and sub-polar areas. A similar distribution is found in all the ocean basins, with varying magnitudes though. Between 40°S to 50°N, the stirring component is 1 to 10 times larger than the trapping component. In the Indian section of the ACC region, however, the trapping heat transport reaches ~0.3 PW, comparable to the stirring component.

This latitudinal dependence of the stirring heat transport is similar to one of the previous estimates that are based on cross covariance method and high-resolution model outputs^4^ (green lines); however, it has larger magnitudes than another^3^ (gray lines). Moreover, the present estimate has different peak locations from the model estimates. Another notable feature is that, the present estimate of trapping component has smaller magnitude and weaker meridional variations than ref.^14^ (yellow line), which is primarily due to the same meridional movement direction of cyclonic and anti-cyclonic eddies. Exception exists in the ACC region where both cyclonic and anti-cyclonic eddies have opposite meridional directions and thus enhance each other in meridional heat transport. Differences in methods may also be part of the sources for the discrepancies between the two estimates: we calculated eddy trapping heat flux and heat transport through building average 3D eddy structures, while ref.^14^ applied a correlation method.

The total eddy heat transport can account much for the estimated total oceanic heat transport^10^ (magenta line) in some latitudes, particularly in the heat convergence zones. The total oceanic heat transport peaks at 1.2 PW and −2.0 PW at 10°N in the northern hemisphere and 20°S in the southern hemisphere, 4 and ~2.5 times as large as the eddy peak transports, respectively (Fig. 4a). Hence, the peaks of the eddy heat transport are about 25–40% of the total. Note that, the latitudes of peak eddy heat transport do not coincide with those of the total transport; rather, they are ~20°−30° poleward of the latter. In particular, at northern and southern locations of the eddy heat transport peaks, the eddy heat transports account for about 50% of the total heat transport, greatly compensating for the large scale circulation component.

## Summary and Discussion

In this study, we figured out the basin-scale distribution of oceanic eddies by reconstructing 254 3D cyclonic and anticyclonic eddies based on millions of Argo profiles, and accordingly estimated the mean meridional eddy heat fluxes and heat transport at 5° × 5° boxes. The composite 3D eddies show great variation with latitudes and longitudes, which are crucial in correctly constructing the spatially varying eddy heat fluxes and transports. Accordingly, some important issues related to the eddy heat transport, such as the pathway of eddy heat transports, the relative importance of eddy heat transports and mean flow heat transports, the relative contribution by the stirring and trapping components of eddy heat transports, and its comparison with model simulations, are identified. The eddy heat transports are confined to mid-latitudes of the western part of ocean basins in the up 1000 m, and are associated with western boundary currents and larger background SST gradients. The total eddy heat transports peak at 0.8 PW and 0.3 PW at about 45°S and 35°N in the southern and northern hemispheres, respectively, accounting for about one half and one third of the total heat transport at the same locations. The stirring component of the eddy heat transport is dominant in the northern hemisphere and in the subtropical to mid-latitudes of the southern ocean; in the ACC region, however, both the stirring and trapping components are comparable in magnitudes.

The present results could serve as useful references for validating and improving both high- and low-resolution models. While the performance of high-resolution models in simulating eddies could be evaluated by comparing their sea surface kinetic energy spectrum with those from the satellite observations, no sufficient data are available for such a comparison below the sea surface. The present 3D distribution of eddy fluxes with both their stirring and trapping components, instead, could be used as reference for subsurface evaluation. On the other hand, the 3D pattern eddy heat could act as the basis in constructing parameterization schemes for eddies’ effect on heat balance in the low-resolution ocean and climate models. Imperfections of existing parameterization for the stirring eddy fluxes^18,19^ are argued to account for part of model bias and should be optimized^20^; and the spatially varying trapping effect, as large as the stirring effect in the ACC, no doubt should be properly parameterized, too.

The previous eddy representative problem was solved to a great extent in this study by reconstructing about two hundreds eddies covering all the latitudes and longitudes, however, uncertainties may remain due to two aspects, all of which are still relevant to insufficiency of data. The first is the sparse Argo profiles in low and high latitudes, where the sub-regions should be zonally elongated to composite eddies. In consequence, we have less capability to solve the latitude dependency of eddies in these regions. Under such conditions, the dipole component of the temperature anomalies may be underestimated during reconstruction of the eddy because of the “average” nature of the method, which, accordingly, may yield underestimated heat fluxes; the problem is only (partly) amended above the mixing layer (see Method) but left unsolved below. The second is from the eddy dataset^17^, which selected only the long lifetime (>4 weeks) eddies and thus exclude the possible effects of short lifetime or shorter scale eddies^2^. In addition, the determination of the eddy trapping depth may also result in uncertainties. Previous study suggested that the eddies can be already leaky above such defined trapping depth^26^. These uncertainties may be identified and fixed in the future when more data and more comprehensive eddy detection method become available. In addition, it should be noted that we have neglected the effects of eddies during their growing and dacaying stages.

## Data

1. A global meso-scale eddy dataset consisting of eddy tracks with eddy lifetime of at least 4 weeks and eddy amplitudes of least 1 cm is used (from http://wombat.coas.oregonstate.edu/eddies/, ref.^17^). Eddies are detected and tracked by an automated algorithm from sequential satellite altimeter sea level anomaly (SLA) fields, spanning from January 2006 to 2015. We select snapshots during the eddies’ stable stage (between 1/5 and 4/5 of their lifetime) for analysis.
2. Argo profiling float data (from http://www.coriolis.eu.org) is applied for compositing 3D eddy structures (only the profiles that are co-located with detected surface eddies are used). The temperature/salinity (T/S) profiles are interpolated from sea surface to 1000 m with spacing width of 10 m, spanning from 2006 to 2015.
3. Daily climatological T/S profiles (Commonwealth Scientific and Industrial Research Organization (CSIRO) Atlas of Regional Seas 2009, CARS09) are used in calculating temperature, salinity and dynamic height anomalies, and hence geostrophic velocities (see below).
4. SST data derived from the Advanced Microwave Scanning Radiometer for Earth Observing System (AMSR-E) sensor onboard the EOS Aqua satellite, with a spatial resolution of 0.25° and ranging from 2002 to 2011, is applied to derive the surface eddy SST anomalies.
5. The Archiving, Validation and Interpretation of Satellite-merged sea surface height (SSH) anomaly (SSHA) product (AVISO; http://www.aviso.oceanobs.com), with a spatial resolution of 1/3° × 1/3° and a 7-day temporal resolution from 2002 to 2011, is used for compositing SSH anomalies, and hence the surface geostrophic currents (surface eddy rotation velocities).

## Methods

### Compositing three-dimensional eddy at sub-regions

The 3D eddy is reconstructed in each sub-region (rules for determining a sub-region are detailed in text) based on concurrent data of sea surface eddy and Argo profiles:

1. The daily climatological T/S profiles provided by CARS09 at corresponding date and location are subtracted from the Argo T/S profiles to obtain temperature and salinity anomalies (*T*′ and *S*′, respectively). Daily climatological dynamic height is also subtracted from the observed dynamic height profile to obtain dynamic height anomalies (*D*′). The dynamic height, relative to 1000 m reference depth, was computed by vertical integration of specific volume anomaly, which is estimated from the temperature and salinity fields.
2. For each snapshot of the surface eddy that is during the stable stage, i.e., between 1/5 and 4/5 of their lifetime, concurrent Argo profiles (those on the same day the eddy has been detected) within two eddy radii are selected. The relative position of each Argo profile to the eddy center is standardized by the eddy radius, and converted into eddy coordinate space (ΔX, ΔY).
3. Rearrange the profiles of anomalies into 0.1° × 0.1° × 10 m grid points within a bin up to a horizontal distance of two eddy radii, by using the inversed distance weighting (IDW) interpolation. For each grid point, Argo profiles locate within the horizontal range of 0.1*R*_0_ are set the weight value
  $${W}_{i}={e}^{-{(d/{R}_{0})}^{2}},$$
  where *d* denotes the distance from the profile to the grid point, and *R*_0_ denotes the eddy radius. The final value at each grid point, ${A^{\prime} }_{grid}$, is calculated from the profile values *A*′ as:
  $${A^{\prime} }_{grid}=\sum {W}_{i}{A^{\prime} }_{i}/\sum {W}_{i}.$$
  *A*′ stands for *T*′ and *D*′.
4. Eddy rotation velocity (geostrophic velocity anomaly) is calculated based on the obtained composite *D*′ as

$${U^{\prime} }_{g}=-\,\frac{g}{f}\frac{\partial D^{\prime} }{\partial y},{V^{\prime} }_{g}=\frac{g}{f}\frac{\partial D^{\prime} }{\partial x}.$$

where *g* is the gravitational acceleration and *f* is the Coriolis parameter.

In addition, in order to calculate heat flux associated with the eddy, the following properties of a composite eddy are also determined:

- Eddy size: defined in terms of sea surface eddy radius (*R*_0_) which is determined by maximum mean rotational geostrophic velocity (Fig. 1).
- Eddy intensity: defined by the maximum eddy rotation velocity at sea surface (Fig. S3c).
- Eddy trapping depth (*TD*): defined as the depth at which the mean eddy rotational velocity equals to the mean movement speed (Fig. 1). Above the trapping depth, eddies scarcely exchange water with its surroundings; in contrast, downward from it eddies lose nonlinearity and hence the ability to trap and transport water properties^11,12^. Therefore, eddy trapping depth can be considered as the eddy thickness. Trapping depth is only adopted in calculating depth-integrated trapping heat flux/transport of the composite eddy.

### Meridional eddy heat flux based on individual composite eddies

Based on the composite eddy, following ref.^8^, we define the mean stirring (*F*_*stir*_) and trapping (*F*_*trap*_) components of the meridional eddy heat flux *across an unit length* at a certain depth *z* as

1.1 $${F}_{stir\_unit}(z)=\frac{1}{4}{\rho }_{0}{C}_{p}{\int }_{-2}^{2}{V}_{g}\text{'}(y=0,z)\cdot T\text{'}(y=0,z)dx,$$

and

1.2 $${F}_{trap\_unit}(z)=\frac{1}{4}{\rho }_{0}{C}_{p}{\int }_{-2}^{2}{V}_{p{5}^{\circ }}\text{'}\cdot T\text{'}(y=0,z)dx,$$

respectively. Here, the calculation is conducted at normalized eddy coordinate where the eddy radius is unit, and the lateral integration extension is set to four normalized eddy radii (i.e., from −2 to 2), because the radial average of the rotation velocity at 2 radii is around zero (Fig. 1). y = 0 denotes the east-west section across the eddy center. Here *V*_*g*_′ and *V*_*p*_′ are meridional geostrophic velocity (eddy rotational velocity) anomay of the eddy and the mean meridional eddy propagation velocity in the sub-region, respectively. The latter is derived from the global eddy dataset^11^. *T*′ is eddy temperature anomaly, *C*_*p*_ is the specific heat capacity (4200 J·kg^−1^·K^−1^) and ρ_0_ is a reference density (1025 kg·m^−3^).

As an example, the stirring and trapping heat fluxes associated with the entire eddy are determined as:

$${F}_{stir\_eddy}(z)=4{R}_{0}{F}_{stir\_unit}(z)$$

and

$${F}_{trap\_eddy}(z)=4{R}_{0}{F}_{trap\_unit}(z),$$

respectively (Fig. S2d).

Both ${F}_{stir\_unit}$ and ${F}_{trap\_unit}$ are assigned to grids within the sub-region where the eddy is composited, and are used for further calculations at the 5° × 5° boxes as described below.

### Composite sea surface eddy at 5° × 5° boxes and compute surface heat fluxes

Because there exist sufficient, high- spatial and temporal resolution sea surface data, in order to take advantage of the abundant sea surface eddy information and to explore smaller (than sub-regional) scale structures of eddy heat transport, sea surface eddies including ${V^{\prime} }_{g}(z=0)$, ${V^{\prime} }_{p}(z=0)$ and *T*′(*z* = 0) are composited in 5° × 5° boxes. Here, ${V^{\prime} }_{g}(z=0)$ and ${V^{\prime} }_{p}(z=0)$ are the surface geostrophic velocity associated with the eddy, calculated from SSHA fields. Both *T*′ and SSHA are the difference between the weekly fields and the corresponding monthly climatology. Detailed procedures include:

1. For each eddy snapshot (during their stable stages), select concurrent SSTA and SSHA fields of the same day and within two eddy radii.
2. Bin the SSTA (SSHA) fields laterally according to distance from the eddy center, normalized by the eddy radius, into 0.1 bins up to a distance of two eddy radii, using Cressman interpolation method. The weight function is
  $${W}_{i}=({L}^{2}-{d}^{2})/({L}^{2}+{d}^{2}),$$
  *L* is assigned a value of 0.1 and *d* represents the distance between the SSTA (SSHA) grid and the grid point to be interpolated. Thus both SSTA and SSHA are composited.
3. Surface geostrophic velocities are calculated from the composite SSHA, which are the eddy rotation velocities. Surface eddy movement speed is the mean of individual eddies in the 5° × 5° box (Figs S3 and 4).
4. Surface eddy heat fluxes are calculated using a same way as (1).

### Eddy heat flux profiles at 5° × 5° boxes

We further divided the 5° wide sub-regions into zonally non-overlapping 5° × 5° boxes, which are the same as used for surface eddy composition, and estimated eddy heat fluxes across the corresponding 5° length, based on both of the eddy heat fluxes from sub-regions and surface eddy heat fluxes from 5° × 5° boxes. The two components are estimated as:

2.1 $${T}_{stir\_5^\circ }(z)=\frac{L}{{R}_{0}}{N}_{e}{I}_{s}{F}_{stir\_eddy},$$

and

2.2 $${T}_{trap\_5^\circ }(z)=\frac{L}{{R}_{0}}{N}_{e}{I}_{t}{F}_{trap\_eddy},$$

respectively. Here, four modulation factors, *L/R*_0_, *I*_*s*_, *I*_*t*_ and *N*_*e*_, have been taken into account. *L* is the zonal length of the 5° × 5° boxes, varying with latitudes. *R*_0_ is the composite eddy radius at the corresponding sub-region. *I*_*s*_ (*I*_*t*_) is the eddy intensity factor, determined as ratio of the surface eddy stirring (trapping) flux of an individual eddy composited at the 5° × 5° box (see Method above) over the mean of the surface eddy stirring (trapping) flux averaged at all 5° × 5° boxes within the corresponding sub-region. Therefore, this factor is used for simply representing the zonal variation of the magnitude of heat fluxes within the sub-region; the structure of the factor is shown in Fig. S5. *N*_*e*_ is the local occurrence frequency factor, which is determined as the accounts of eddy snapshots in each 5° × 5° box multiplied by the eddy sampling interval (1 day) and divided by the research period. The global distribution of it is shown in Fig. S5. It is also seen that, even within a same sub-region, *N*_*e*_ is different from box to box. Consequently, the factors convert the estimated heat flux of an individual eddy into the time-mean heat flux modulated by eddy intensity and concentrations^14^.

### Matching surface eddy flux and subsurface eddy flux

We note that there exist differences between the satellite data-based surface heat flux and the Argo data-based heat flux near the surface layers at some sub-regions, which is identified to be related to the difference in the composite eddy temperature structures: the latter has less obvious dipolar temperature anomalies than the former. This is because the temperature anomalies in the mixed layer include not only information of geostrophic mesoscale eddies, but also that of ageostrophic, sub-mesoscale and Ekman processes. Those signals can only be eliminated by sufficient Argo profiles. In contrast, below the mixed layer, the geostrophic processes are dominant and the eddies can be more easily reconstructed. We fixed the mixed layer inaccuracy problem by matching the satellite data-based surface heat flux and the Argo data-based heat flux at the bottom of mean mixed layer, via interpolating (the mixed layer depth is obtained from climatology). Firstly, at each 5° × 5° box, the value of the satellite data-based heat flux is specified as the eddy heat flux at the surface; and then, based on the surface value and the Argo-data-based values below the mixed layer, the values in the mixed layer is interpolated with a cubic method.

### Vertically integrated meridional heat transport of a composite eddy

The two components of depth-integrated heat transport at a 5° × 5°box are calculated as

$${Q}_{stir\_5^\circ }={\int }_{1000}^{0}{T}_{stir\_5^\circ }(z)dz$$

and

$${Q}_{trap\_5^\circ }={\int }_{TD}^{0}{T}_{trap\_5^\circ }(z)dz,$$

respectively. Here, *TD* denotes the eddy trapping depth of corresponding sub-region. Because no theory has suggested a specific depth for eddy stirring effect, and stirring heat flux by a composite eddy usually decays to ~0 before it reaches 1000 m, we adopted the upper 1000 m as the depth of effective stirring. Our results suggest that this treatment is appropriate since contributions below 1000 m are small in most of the global oceans.

## Supplementary information

supplementary figures
