# Supplementary material for: Global meridional eddy heat transport inferred from Argo and altimetry observations
Source: Sci Rep. 2019 Feb 4;9:1345. doi: 10.1038/s41598-018-38069-2 (PMC6361900; doi:10.1038/s41598-018-38069-2)
Supplement: Supplementary file 1 — supplementary figures [file 41598_2018_38069_MOESM1_ESM.doc]

Global meridional eddy heat transport inferred from Argo and altimetry observations

Bowen Sun^1,2^, Chuanyu Liu^1,3,4,*^ Fan Wang^1,3,4,*^

^1^ CAS Key Laboratory of Ocean Circulation and Waves, Institute of Oceanology, Chinese Academy of Sciences (IOCAS), Qingdao 266071, China

^2^ University of Chinese Academy of Sciences, Beijing, China

^3^ Marine Dynamic Process and Climate Function Laboratory, Pilot National Laboratory for Marine Science and Technology (Qingdao) (QNLM), Qingdao 266237, China

^4^ Center for Ocean Mega-Science, Chinese Academy of Sciences, Qingdao 266071, China

*Correspondence and requests for materials should be addressed to C.L. (email: chuanyu.liu@qdio.ac.cn) or F.W. (email: fanwang@qdio.ac.cn)


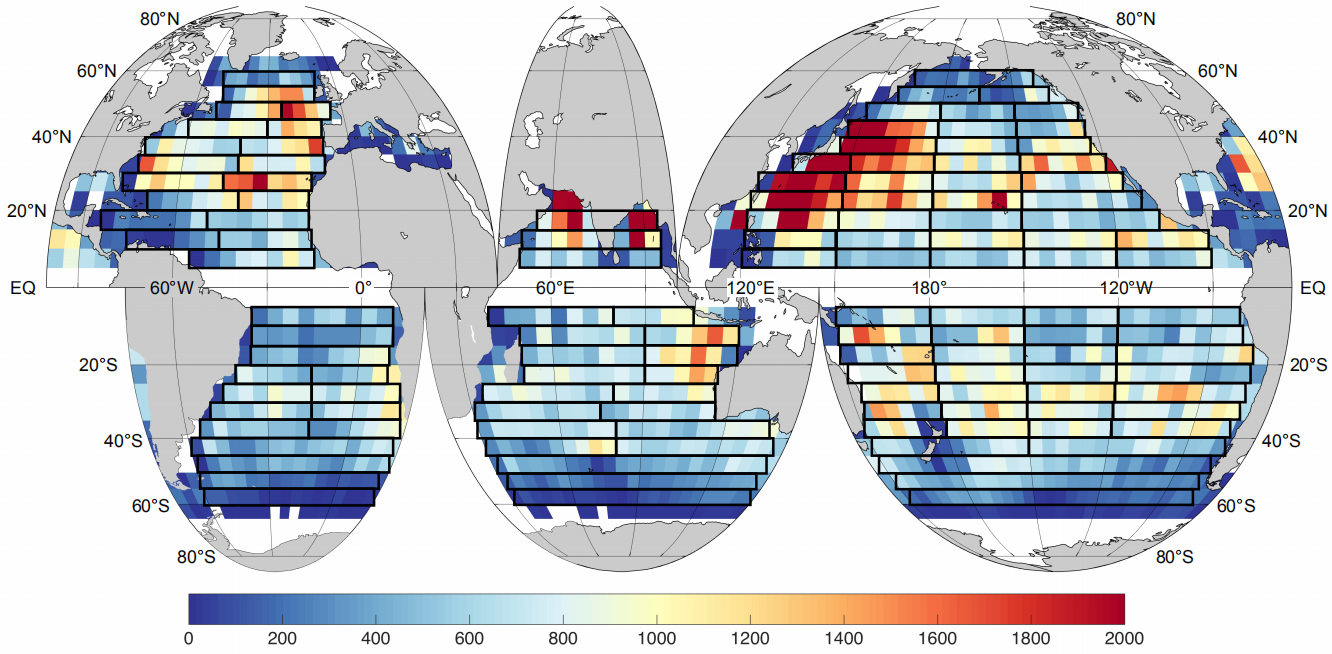


**Figure S1 | Population of Argo profiles concurrent with eddies.** Color shading: number of Argo profiles trapped inside two eddy radii in 5°×5° boxes. The 127 sub-regions are shown bounded by black grids.


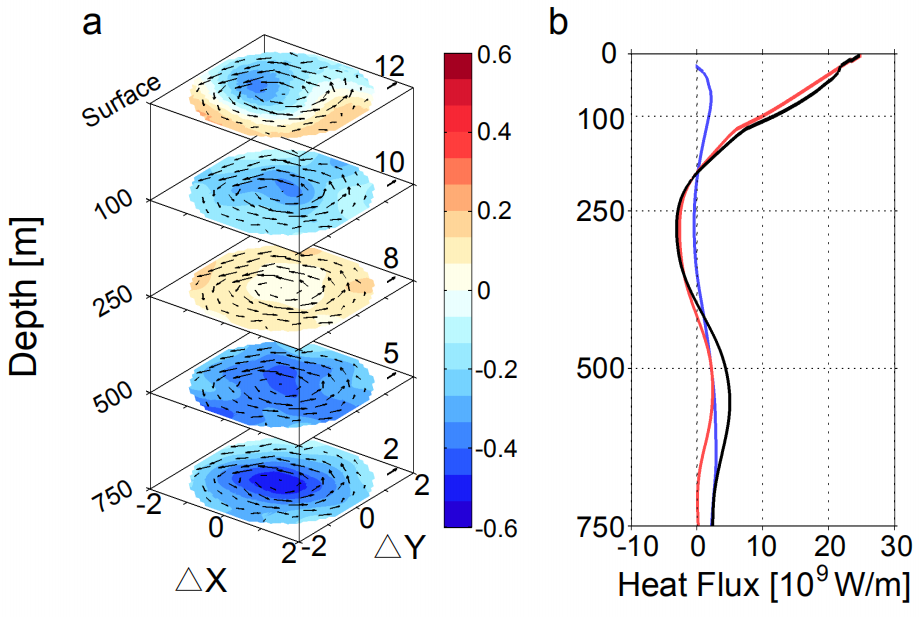


**Figure S2 | A composite cold-core (cyclonic) eddy and associated fluxes in the northern Pacific.** (a) Eddy with anomalous temperature (color shading, in °C) and geostrophic rotation velocity (vectors, in cm/s) at sub-region of 25°N-30°N, 120°E-150°E. Note that the top level is derived from the composite sea surface eddy at the same region. Also note that the reference vectors vary with depth levels. (b) Vertical profiles of trapping (blue), stirring (red) component and total (black) heat flux.


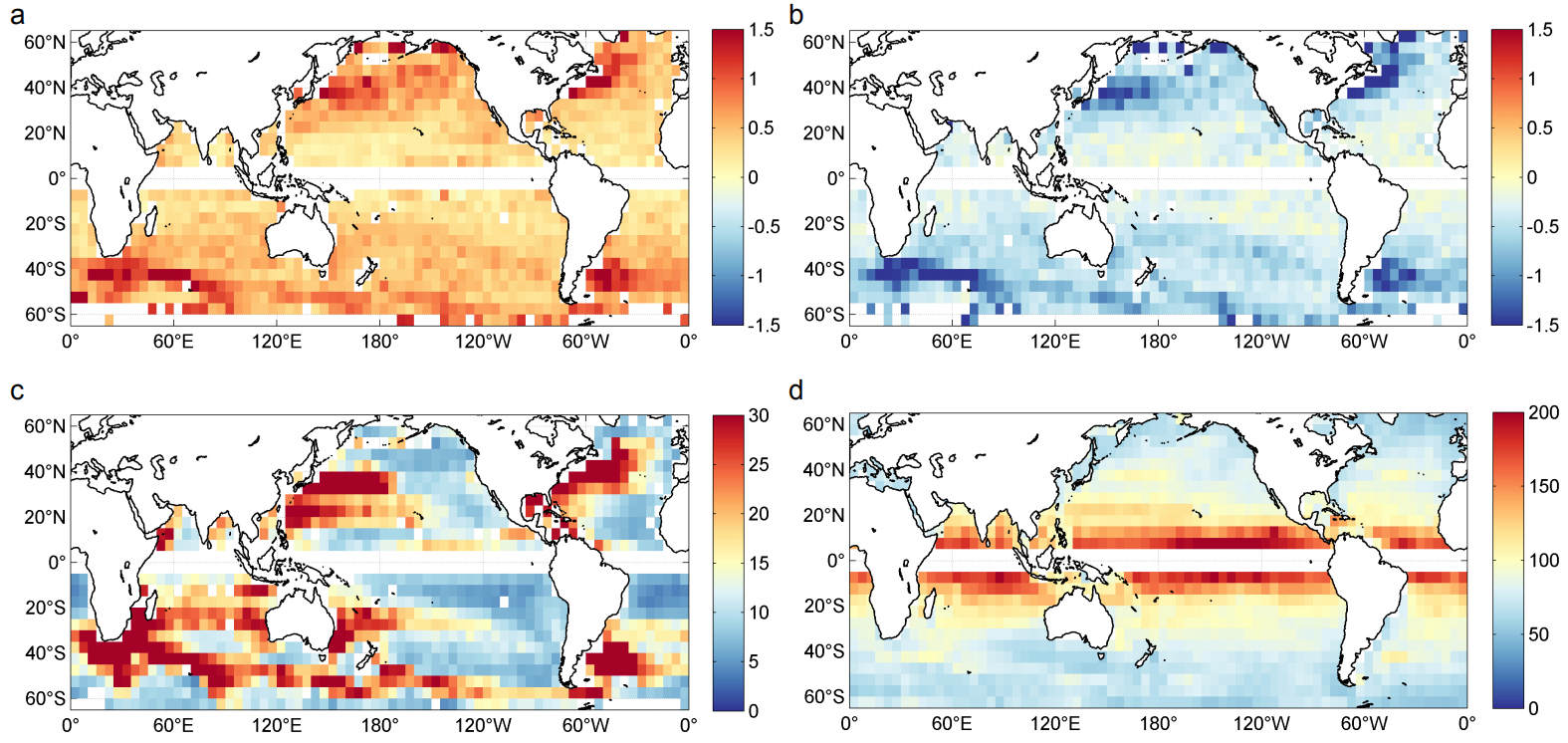


**Figure S3 | Characteristic eddy properties.** Shown are maximum anomalous SST, *T'(z=0*) (in °C) associated with (a) warm-core (anti-cyclonic) and (b) cold-core (cyclonic) sea surface eddies, (c) maximum sea surface geostrophic rotation velocity of both types of eddies (in cm/s), and (d) average radius of the eddies (in km). All properties are calculated at 5°×5° boxes.


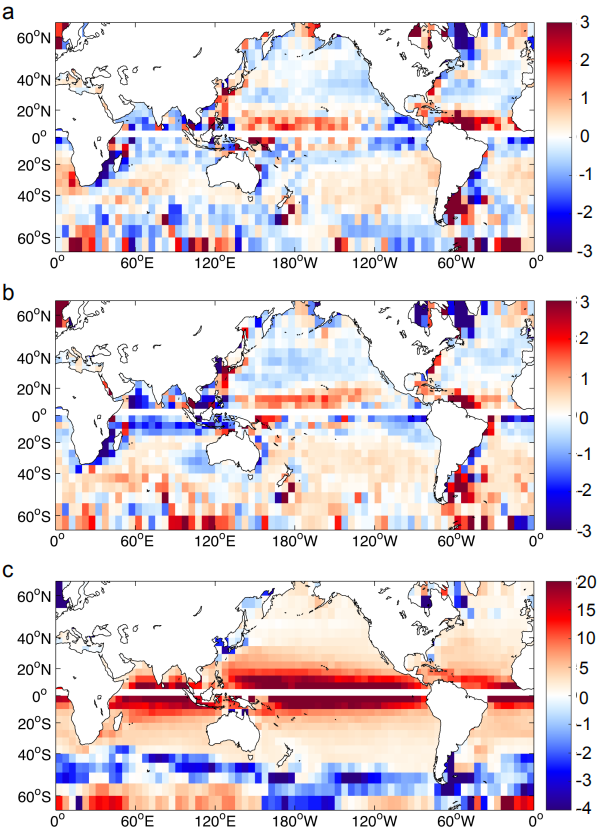


**Figure S4 | Propagation speed of eddies.** Shown are mean northward propagation speed of (a) anti-cyclonic and (b) cyclonic sea surface eddies, and (c) westward propagation speed of both types (unit: cm/s). All properties are averaged in 5°×5° boxes.


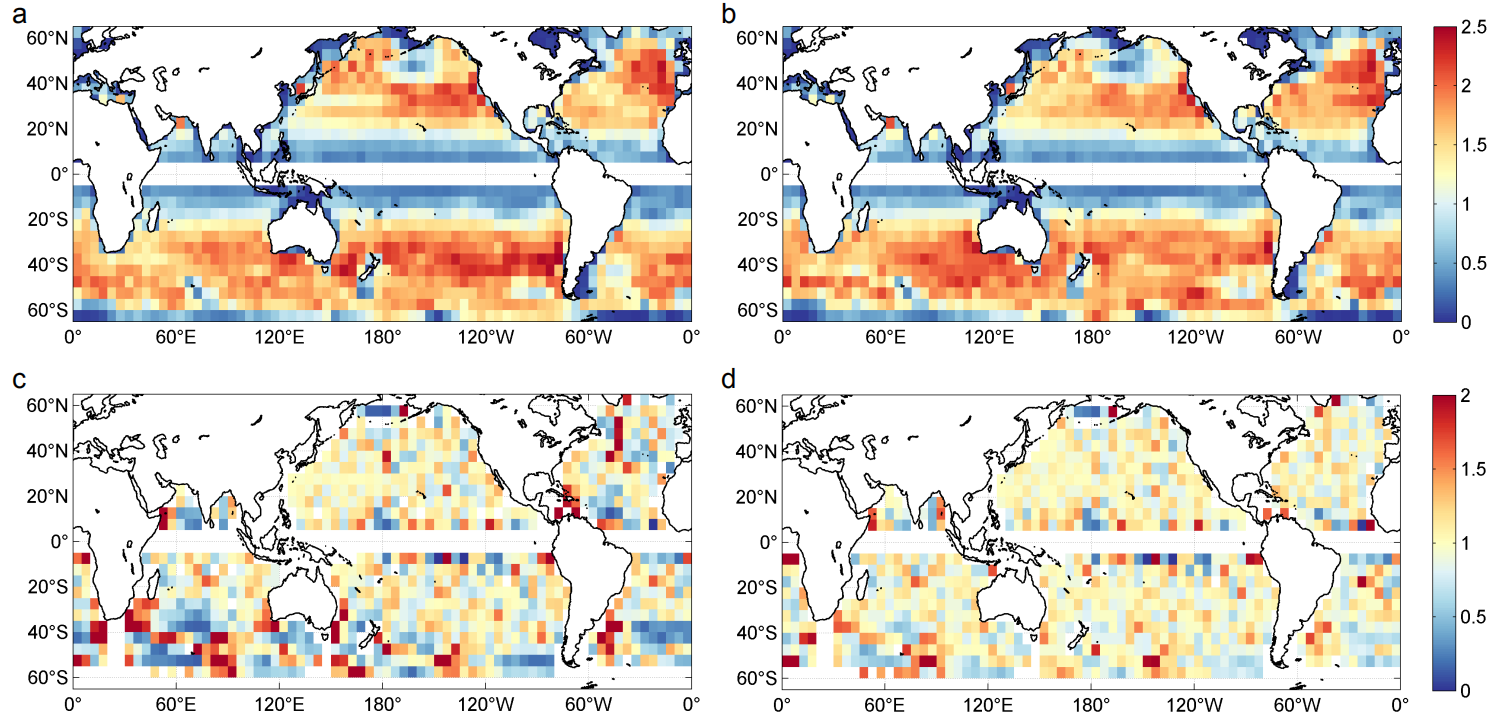


**Figure S5 | Eddy occurrence frequency (*N_e_*) and intensity factors (*I_t_*, *I_s_*).** Shown are *N_e_* for (a) anti-cyclonic and (b) cyclonic sea surface eddies, respectively, and eddy intensity factor for (c) trapping (*I_t_*) and (d) stirring component of eddies(*I_s_*), respectively. All properties are calculated at 5°×5° boxes.


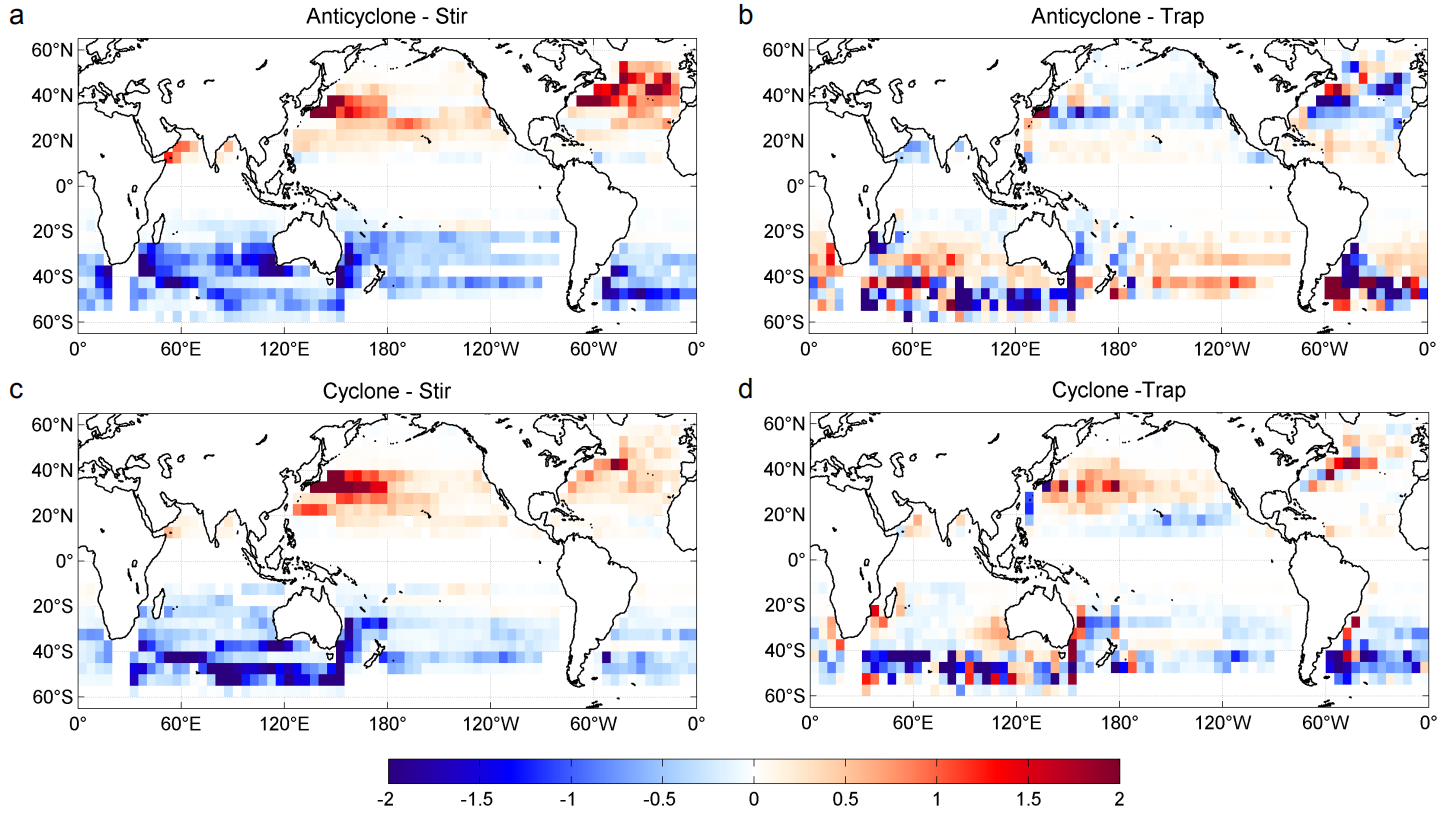


**Figure S6 | Components of the meridional eddy heat transport.** Shown are depth and zonally integrated meridional eddy stirring (a, c) and trapping (b, d) heat transport (unit: 10^13^ W) induced by cyclonic (c, d) and anti-cyclonic (a, b) eddies 5°×5° boxes. Zonal integration is over 5° length (for each 5°×5°). Positive and negative values denote northward and southward heat transport, respectively.
